# Mixing Languages during Learning? Testing the One Subject—One Language Rule

**DOI:** 10.1371/journal.pone.0130069

**Published:** 2015-06-24

**Authors:** Eneko Antón, Guillaume Thierry, Jon Andoni Duñabeitia

**Affiliations:** 1 BCBL, Basque Center on Cognition, Brain and Language, Donostia, Spain; 2 School of Psychology, Bangor University, Gwynedd, Wales, United Kingdom; University of Barcelona, SPAIN

## Abstract

In bilingual communities, mixing languages is avoided in formal schooling: even if two languages are used on a daily basis for teaching, only one language is used to teach each given academic subject. This tenet known as the one subject-one language rule avoids mixing languages in formal schooling because it may hinder learning. The aim of this study was to test the scientific ground of this assumption by investigating the consequences of acquiring new concepts using a method in which two languages are mixed as compared to a purely monolingual method. Native balanced bilingual speakers of Basque and Spanish—adults (Experiment 1) and children (Experiment 2)—learnt new concepts by associating two different features to novel objects. Half of the participants completed the learning process in a multilingual context (one feature was described in Basque and the other one in Spanish); while the other half completed the learning phase in a purely monolingual context (both features were described in Spanish). Different measures of learning were taken, as well as direct and indirect indicators of concept consolidation. We found no evidence in favor of the non-mixing method when comparing the results of two groups in either experiment, and thus failed to give scientific support for the educational premise of the one subject—one language rule.

## Introduction

Although some of the positive consequences of bilingualism in domain-general cognition [1–3] remain debated on the basis of data showing similar performance in bilinguals and monolinguals in executive control tasks [4–6], benefits of bilingualism at a linguistic level seem to be less controversial and appear generalizable. For instance, bilinguals have been shown to outperform monolinguals in phonetic awareness tasks [7] or new vocabulary acquisition [8]. The positive–linguistic–consequences of bilingualism are well-accepted, and the negative impact of early bilingual immersion is at the very least debatable, considering that bilingual children have been shown to reach the same linguistic milestones as monolinguals over the same developmental periods [9–10].

It is a widely held view in bilingual education that introducing more than one language “too early” in life may be detrimental to learning by delaying language acquisition or even triggering confusion between languages in children. However, scientific observations show that children can learn more than one language in a naturalistic context in a seemingly effortless way, and there is little evidence to date of a detrimental effect produced by bilingual education (see [11] for a detailed description of this “bilingual paradox”). In fact, a number of studies have reported an advantage in bilinguals who are exposed to (and use) two or more languages from birth as compared to late bilinguals, since early bilinguals usually show greater fluency and mastery in almost every aspect of their second language (L2) [12–15]. More importantly for the purposes of the current study, it has been shown that children immersed in a bilingual educational context learn new words better than children immersed in a monolingual context [16].

Given the prevalence of bilingualism in modern societies and the multiplication of policies advocating the protection of minority languages [17], the inclusion of bilingualism in education is a key issue in regions where two or more languages have equal official status (e.g., Catalonia or the Basque Country, which hold Catalan or Basque, respectively, to an equal status as Spanish, or Wales, where Welsh is the official language on a par with English). This also happens in places where a new language is progressively developing (as indexed by the increasing number of speakers) as is the case for Spanish in the United States [18]. In these circumstances, the two languages of a bilingual community tend to be represented in the educational system. While there are different ways in which bilingual education can be implemented, one of the most widespread methods is the Two-Way Immersion program (TWI) [19, 20]. The TWI promotes the use of the two languages as vehicular languages, and it has been adopted in most countries with strong bilingual communities. This method has been implemented either on the basis of 50/50 exposure (i.e., children receive instruction and tuition half of the time in one language and the other half in the other), or on the basis of 90/10 exposure (i.e., children initially receive most of the tuition in the “new/incoming language” and get increasingly exposed to the strongest language, generally aiming to reaching the 50/50 exposure ratio by grade 5) [21].

This being said, it does not seem to matter which method of immersion is employed by a given bilingual school, a core principle prevails: the *one language-one subject rule*. In the vast majority of bilingual schools throughout the world, each subject is taught in a unique language during the whole academic year, and language mixing is avoided within the context of a subject because it is taken for granted that mixing languages would lead to confusion and hinder learning. For illustration purposes, considering a Spanish-English bilingual school, if a given group of students is taught Geography in English and Mathematics in Spanish, English would not be used or allowed during the Mathematics lessons, and Spanish would not be used during the Geography lessons. However, such a radical division is rather unrealistic when taking into account bilingual exposure outside the classroom, given that switching from one language to the other is a highly common behavior in bilingual societies [22–25], and that language switching spontaneously occur from early childhood [26]. Hence, bilinguals receive and transmit information in a language-mixed fashion without effort, but in sharp contrast, it is the single-language context instead of a dual-language context that bilinguals encounter during formal schooling in bilingual schools. The reason behind this one subject-one language rule seems to stem from fears of the detrimental consequences of mixing languages (i.e., the worry that it may lead to confusion when acquiring new concepts and therefore to deteriorate concept acquisition or learning). To the best of our knowledge, however, this commonly held view has not yet received any scientific validation or support. On the contrary, it has been suggested that the consequences of being immersed in a bilingual learning context are potentially beneficial instead of detrimental. In a study with a large sample of Spanish-speaking English learners, Baker and colleagues [27] investigated how participants differed in their English reading achievement depending on the reading teaching methods. They contrasted a single-language (English-only) program and a mixed-language (bilingual) program. The authors found that participants following the mixed-language bilingual approach showed highly similar reading achievement as participants in the single-language group, and that the differences between groups, if any, were in favor of the mixed-language context.

Here, we address this question directly: Is language-mixing during a learning procedure detrimental to learning? In other words, is learning in a mixed-language context less efficient than in a single-language context? Learning, defined here as the acquisition, understanding and retention of new information, occurs spontaneously and very early on in life. But, once children acquire the ability to use language (comprehend and produce utterances), and especially when they start conventional education, learning shifts toward concept acquisition mediated by language. For instance, when encountering the biological definition of ‘heart’, a student may construct her concept from “*something inside that makes you live and love*” or *“a hollow muscular organ that pumps the blood through the circulatory system by rhythmic contraction and dilation”* (from the Oxford dictionary).

Concept learning in monolingual contexts (e.g., how new concepts are recognized, assigned meaning, and consolidated either in L1 or in L2 without language mixing) has been extensively studied over the past decade [28]. Language-mediated learning can be investigated in many different ways, ranging from experimental methods that emulate the moment in which a word is encountered for the first time and its meaning needs to be inferred from context [29] to methods that are based on providing the exact meaning of a new word through exposure to its definition(s) [30]. In the current study, we thus chose to use the inferential learning method (i.e., provide features that characterize a concept instead of merely mapping a name to a particular concept) in order to test whether semantic representations acquired in a mixed-language context differ in quality from those acquired in a monolingual context. The selection of the inferential learning method relies on recent evidence that this method allows to generalize and acquire more stable semantic representations as compared to alternative mapping methods [31].

We investigated whether concepts learnt in a single-language context are better acquired and consolidated than concepts learnt in a mixed-language (i.e., bilingual) context, or–alternatively and in contrast to common belief–whether there is no learning deficit associated with a bilingual learning context. In a mixed-language context, information needs to be decoded in two languages before it is integrated at a common semantic level. Under these conditions, the learning process may be expected to suffer given the additional effort required to switch between languages. However, fluent bilinguals have been shown to spontaneously and unconsciously translate input from one language into their other language [32–38], and several studies have shown that the cost associated with implicit translation is minimal for relatively balanced bilinguals [39–41]; note that this is also the case for unbalanced bilinguals, who manifest sizeable translation priming effects from L2 to L1; [42]. Thus, it could be envisaged that language mixing does not affect learning significantly, given that inputs from the two languages are automatically translated into the other language thus favoring parallel semantic access in highly proficient or balanced bilinguals [43–45].

In Experiment 1, two groups of adult balanced bilinguals were exposed to a concept learning phase either in a single- (monolingual) or in a mixed- (bilingual) language context. We opted for naturalistic learning involving the association of semantic features with a novel unknown visual object (i.e., the inferential learning method; see [31]). One group of participants learnt these concepts in a single-language context in which two features of the object were provided in the same language. The other group of participants acquired these concepts in a mixed-language context, with the two definitions presented in different languages. After the learning phase, participants were tested in a series of experimental tests aimed at quantifying the extent to which semantic acquisition and representation differed across groups. Both direct and indirect measures of concept acquisition were obtained. In Experiment 2, two groups of bilingual children attending a bilingual school were tested using the same experimental paradigm in order to test the extent to which the results in adults would apply to an educational context.

If the one subject–one language rule has any grounding, learning should be better established in the single-language context (SLC) than in the mixed-language context (MLC), given the possible confusion caused by language mixing. If so, enhanced consolidation in the single-language context should be reflected by better performance in tasks directly or indirectly measuring learning and consolidation. If, on the contrary, participants in the single-language context do not outperform mixed-language context learners, then it would be reasonable to call into question the one subject-one language rule, and maybe think of it as a prejudice that has developed on the basis of ill-formed intuition surrounding the bilingual paradox.

## Experiment 1: Adults

### Methods

#### Ethics Statement

All the participants signed informed consent forms before the experiment and were appropriately informed regarding the basic procedure of the experiment, according to the ethical commitments established by the BCBL Scientific Committee and by the BCBL Ethics Committee that approved the experiment (Approval date: 19/03/2014; Approval reference: 19314J).

#### Participants

Fifty young adults (28 females, mean age of 22.96 years) took part in the experiment. All of them were Basque-Spanish balanced bilinguals who acquired both their languages before the age of 6. Their language proficiency in Basque and Spanish was assessed in two ways. First, participants were asked to name a set of 77 common objects in the two languages (see [46] for a similar approach), which showed good vocabulary knowledge in both languages (74.3, SD = 0.82, in Basque and 76.54, SD = 0.08, in Spanish). Second, all participants were individually interviewed by a native Basque-Spanish bilingual linguist in order to assess their communicative skills in each language. The interview started by asking participants to provide basic sociodemographic information, continued with questions related to participants’ personal interests, and ended with questions about how they got to know the research center. The language of the interactions was changed from one question to another so that the two critical languages could be assessed in detail. After each interview, the linguist rated the participant based on his/her performance following a 1-to-5 scale (where 5 represents native-like competence and 1 corresponds to an extremely basic or no knowledge of the language). All participants got scores of 5 in both languages. Participants were assigned to two context groups: the single-language context (SLC) or the mixed-language context (MLC). To control for between-group homogeneity, we made sure that the participants in the two context groups were matched for age, gender, age of acquisition, and proficiency in both Basque and Spanish (all *p*s>.12; see Table 1).

**Table 1:** Controlled variables in the adult samples tested in Experiment 1.

	Age (years)	Gender (females)	IQ (correct responses)	Flanker Effect (ms)	Simon Effect (ms)	Spanish AoA (years)	Basque AoA (years)	Spanish Vocabulary (out of 77)	Basque Vocabulary (out of 77)
MLC	22.92 (3.12)	56%	23.76 (2.73)	34 (21)	48 (32)	0.76 (1.45)	0.4 (0.87)	76.6 (0.71)	73.72 (3.22)
SLC	23 (2.94)	56%	24.72 (1.72)	44 (28)	36 (22)	1.08 (1.8)	0.36 (1.04)	76.48 (0.92)	74.88 (2.54)
P-value	0.92	1	0.22	0.21	0.21	0.55	0.89	0.66	0.12

Means are presented together with the standard deviations (in parentheses).

In order to ensure that participants in both groups did not significantly differ in terms of domain-general cognitive abilities, three experimental tasks were designed for matching purposes. The first task comprised an assessment of participants’ non-verbal IQ obtained from an abridged version of the Kaufman Brief Intelligence Test, K-BIT [47]. Participants had a maximum of 6 minutes to correctly respond to as many trials as they could from the original set of 34 multiple-choice items. The second task was a classic flanker task [48] consisting of a total of 48 trials, which could be congruent, neutral or incongruent (16 items each). The third task was a Simon task [49], which was also made of 48 congruent, incongruent, or neutral trials (16 items in each condition). These two latter tasks were used to measure participants’ inhibitory skills and to minimize any potential influence of executive control differences on the critical experiments. Pairwise comparisons revealed no significant differences between groups in all three tasks, and the classic indices associated to the flanker and Simon tasks did not differ across the SLC and MLC (all *p*s.>.20, see Table 1).

#### Materials

A set of 40 pictures of unfamiliar tools was selected. These were the unknown objects participants had to learn. Each object was paired with two definitions of well-known daily-life objects (e.g., a key). For instance, the definitions “it is kept in the pocket” and “it unlocks doors’ locks” referring to the common object “key” were associated with one of the novel objects to be learnt (see [31]). In a norming test run during the material creation phase, both definitions were rated for their informativeness (i.e., how well each of the definitions matched the real object they were derived from) and results showed that the definitions were highly informative, with a mean rating of 4.16 out of 5 (SD = 0.91). Also, we avoided prevalence of one definition over the other and we made sure that each definition of a pair was equally informative about the object (*p*>.81). For the MLC, one definition in each pair was translated to Basque (see S1 Appendix for the complete set of definitions). Informativeness of the definitions was also rated as being highly similar across the two languages in a norming study. Basque definitions had a mean informativeness rate of 4.10 with a SD of 0.98, while Spanish definitions had a mean informativeness rate of 4.22 and an SD of 0.85 (p>.53).

#### Procedure

**Fig 1:**
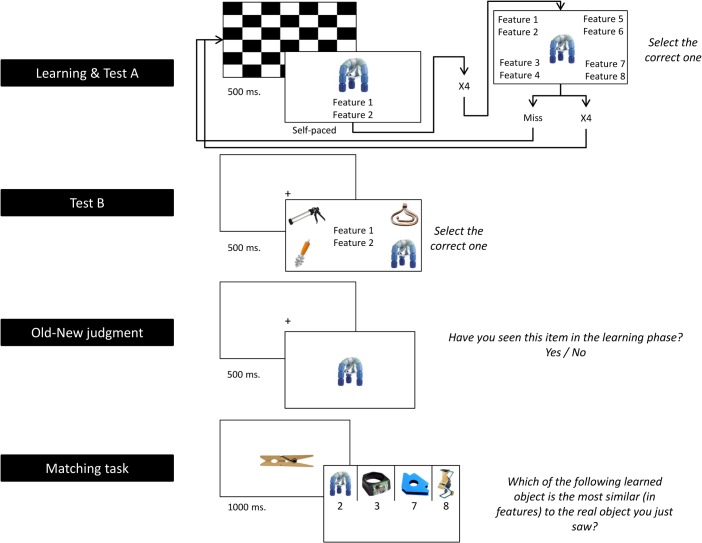
Schematic representation of the Experiments.

The whole experimental session lasted for about one hour in total (see Fig 1 for a schematic summary of the procedure). After the three short control tasks used to match the groups (IQ test, flanker task and Simon task), the learning phase started. Participants learnt the new objects in blocks of four. The pictures were presented one-by-one in the middle of a screen with two features written below them. Learning was self-paced: When a participant thought he/she had learnt the object and its features, he/she could move to the next trial by pressing the spacebar. After every block of four trials, they were tested on the items of that block in order to get an estimate of their immediate learning (Test A). In Test A, one of the learnt pictures appeared in the center of the screen, surrounded by 4 written feature pairs. One of the feature pairs was the correct one, while the others were distractor pairs corresponding to other objects learnt in the same session. If participants failed any of the 4 trials, they had to repeat the whole block of trials and retake Test A, until they succeeded in all 4. Once they met this criterion, the learning session moved to the next block of 4 items. Thus, they went through 40 items in total. Each item appeared twice over the entire learning session (to counterbalance definition order). Both participant groups learnt exactly the same objects, either with the two definitions in Spanish (SLC group), or with one definition in Spanish and the other in Basque (MLC group).

After the learning phase, a short association test started (Test B). In each trial of Test B, participants read a feature pair (i.e., the definitions) displayed on the middle of the screen and were instructed to select the corresponding object from 4 pictures presented on the screen. In Test B, participants responded to the 40 items in a row, no feedback was given on the response accuracy and errors did not trigger a test repeat. This test was used to assess immediate recall after the initial learning phase, and taken as an index of learning.

After completion of the learning phase (Test A and Test B), participants completed an **Old-New judgment task**. After a fixation point (centrally displayed for 500 ms), participants were presented with a target picture in the middle of the screen for a maximum of 3000 ms or until a response was given. Targets consisted of the 40 learnt unfamiliar objects (the *Unfamiliar Old* items) intermixed with 40 unfamiliar objects they had not learnt (*Unfamiliar New* items), 40 familiar objects (*Familiar New* items), and the 40 familiar objects from which the definitions of the learnt objects were derived (*Familiar Related* items; e.g., the picture of a real key). All the images are presented in S2 Appendix.

Eighteen participants who did not take part in the experiment (9 females) rated the 160 items for their familiarity on a scale from 1 to 7 (1 = highly unfamiliar; 7 = highly familiar). This was done in order to ascertain that the objects in the *Unfamiliar New* and *Unfamiliar Old* conditions did not differ from each other in terms of familiarity, and that the objects used in the *Familiar New* and *Familiar Related* conditions were also equally familiar. An unifactorial ANOVA was run on the results, showing significant differences across conditions (*F*(3,51) = 70.43, *p*<.01). Pairwise comparisons demonstrated that the items in the two *Unfamiliar* conditions did not differ from each other (*Unfamiliar New* = 2, *Unfamiliar Old* = 1.95; *t*(34) = -.17, *p*>.86). Similarly, the objects in the two *Familiar* conditions did not differ for each other (*Familiar Unrelated* = 6.12, *Familiar Related* = 6.24; *t*(34) = .25, *p*>.80). As expected, the *Familiar* conditions significantly differed from the *Unfamiliar* conditions (all *t*s>10 and *p*s<.01).

Participants were asked to respond as fast and as accurately as possible by pressing one out of two buttons on a response box to determine whether the displayed objects corresponded to the learnt objects (“Old” items; *Unfamiliar Old* condition) or to any other object not displayed during the learning phase (“New” items; *Unfamiliar New*, *Familiar New* and *Familiar Related* conditions). No feedback was provided to participants during the task. Accuracy rates as well as reaction times were collected.

*Familiar New* and *Familiar Related* items were included in order to have a direct measure of false memory effects. The false memory effect is a well-studied phenomenon consisting of participants showing impoverished performance in identifying that they have not previously seen a concrete item (for example, the word “sleep”) when there is a close semantic relationship between this item and others from the study set (for example, “bed”, “night”, “dream”; see, among many others [50–52]). Hence, the false memory effect is calculated by contrasting the RTs and error rates in the *Familiar New* and *Familiar Related* conditions. *Unfamiliar New* items were included in order to avoid any possible response bias due to a different proportion of *Familiar* and *Unfamiliar* materials. As seen, the inclusion of the necessary control conditions (*Familiar New* and *Unfamiliar New*) makes the proportion of expected “Old” and “New” responses different from the 50%-50% ratio used in some paradigms. However, it should be considered that this relative unbalance is rather usual in the memory literature on the false memory effect [52,53].

A final **Matching task** was also administered to the participants in order to explicitly measure the association strength between the learnt objects and their familiar associates (e.g., between the learnt object that corresponded to a tool that can be kept in the pocket and that is used to unlock doors, and a real key). Participants were presented with the items used in the *Familiar Related* condition from the Old-New judgment task (e.g., the picture of a key) for 1000 ms, followed by the presentation of 4 different objects from the learning phase (i.e., the *Unfamiliar Old* items) on the upper part of the screen. From left to right, each of the 4 target objects was associated with a specific button from a response box, and participants had to indicate as accurately as possible which of the objects was the closest in meaning to the reference stimulus (e.g., identify which of the *Unfamiliar Old* objects was conceptually similar to a real key). Items remained on the screen until a response was given and there was no time limit. Considering that participants needed to simultaneously recall the definitions associated with the 4 learnt items displayed and check which of them best matched the real object presented, accuracy only was used as a dependent measure in this task.

## Results

### Learning (Test A and Test B)

The two groups of participants displayed a similar learning trend (see Table 2 for detailed results of all the tasks). Error rates did not differ between groups in Test A (mean error rates of 2.4% and 2.9% for the MLC and SLC groups, respectively; *t*(48) = -.47, *p*>.64), suggesting that immediate learning did not differ between the SLC group and the MLC group. Similarly, the two groups saw a similar number of items (including block repetitions after a given error; *t*(48) = -.47, *p*>.64).

**Table 2:** Mean reaction times and error rates for all the experimental tasks used in Experiment 1.

		Learning—Test			Old-New judgment task										Matching
		Test A		Test B	Unfamiliar Old		Familiar New		Familiar Related		Unfamiliar New		False Memory Effect		
		Items seen	Error Rate	Error Rate	RTs	Error Rate	RTs	Error Rate	RTs	Error Rate	RTs	Error Rate	RTs	Error Rate	Error Rate
MLC	Mean	83.84	2.4	0.3	649	3.4	585	0	613	0.9	656	0.3	28	0.9	2.5
	SD	4.96	3.1	0.83	91	4.01	99	0	97	2.38	123	0.83	22	2.38	3.06
SLC	Mean	84.64	2.9	0.2	653	4	579	0	611	1.3	645	0.2	32	1.3	1.9
	SD	6.9	4.3	0.69	63	4.51	60	0	63	1.93	80	0.69	23	1.93	2.31

In Test B, SLC and MLC groups did not differ in the number of incorrect responses (*t*(48) = .46, *p*>.65), again showing a highly similar performance during the learning process and immediate recall.

#### Old-New judgment task

In the critical Old-New judgment task, we first explored whether SLC and MLC groups differed in their identification of the learnt objects (*Unfamiliar Old* condition). Response times associated with correct responses did not differ as a function of the context to which participants were assigned (*t*(48) = -.17, *p*>.86). Similarly, the two groups did not differ in their accuracy in identifying *Unfamiliar Old* items (*t*(48) = -.50, *p*>.62).

Next, ANOVAs were performed on the response times and error rates associated to each of the three conditions requiring a “New” response (i.e., *Familiar New*, *Unfamiliar New* and *Familiar Related* conditions) following a 3*2 design in which the 3-levels factor Condition was within-participant and the 2-level factor Learning Context was between-participants factor. The ANOVA on the RTs showed a main effect of Condition (*F*(2,96) = 65.43, *p*<.01) but no effect of Learning Context, nor an interaction between the two factors (all *F*s<1). The ANOVA on the error data also showed a significant main effect of Condition (*F*(2,96) = 10.19, *p*<.01), but not effect of Learning Context neither an interaction between the two factors (all *F*s<1).

**Fig 2:**
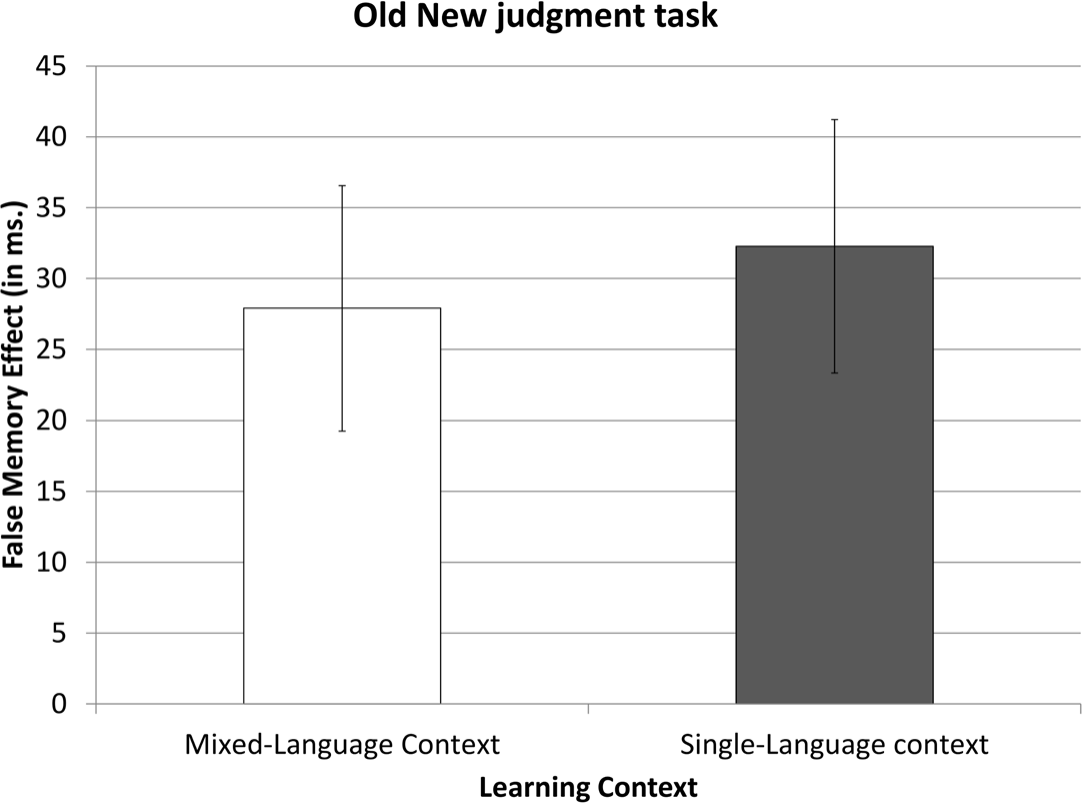
False memory effect in the Old-New judgment task in Experiment 1. This effect results from the subtraction of the reaction times to the Familiar New items from the Familiar Related items. Error bars represent confidence intervals of 95%.

In order to assess concept consolidation, we looked at the false memory effect (i.e., the difference between *Familiar New* and *Familiar Related* conditions). As shown by the results of the t-test on the RTs, *Familiar Related* items were responded to significantly slower than *Familiar New* items (612 ms vs. 582 ms, respectively; *t*(49) = 9.53, p<.01). This 30-ms difference corresponds to the false memory effect elicited by the conceptual overlap between the *Familiar Related* items and the *Unfamiliar Old* items. Critically, the magnitude of this effect was similar in the SLC and MLC groups (effects of 32 ms and 28 ms, respectively; *t*(48) = -.69, *p*>.50; see Fig 2). When looking at the false memory effect on the error data, we also found a significant effect of Condition (*t*(49) = 3.61, p<.01), such that participants made more errors in the *Familiar Related* condition than in the *Familiar New* condition (a 1.1% difference). Participants had difficulty in identifying as “New” the items in the *Familiar Related* condition, given the shared conceptual features with the *Unfamiliar Old* items. As in the RT data, the false memory effect in accuracy was indistinguishable in the SLC and MLC groups (1.3% and 0.9%, respectively; *t*(48) = -.65, *p*>.52).

Mean RTs associated with the *Unfamiliar New* items and those associated with the *Familiar New* and *Familiar Related* items we compared in order to obtain an estimate of the familiarity effect. In line with the difference in familiarity between the *Familiar* and *Unfamiliar* items (see above), results showed that the items in the *Unfamiliar New* condition elicited longer RTs than the items in the *Familiar New* condition (651ms vs. 582 ms; *t*(49) = 10.02, *p*<.01), and than the items in the *Familiar Related* condition (611 ms; *t*(49) = 5.45, *p*<.01). Finally, error rates in the *Unfamiliar New* and the two *Familiar* conditions were compared. *Unfamiliar New* items elicited more errors that *Familiar New* items (0.25% vs. 0%; *t*(49) = 2.33, *p*<.03), but less errors than the *Familiar Related* condition (1.1%; *t*(49) = -2.84, *p*<.01).

#### Matching task

**Fig 3:**
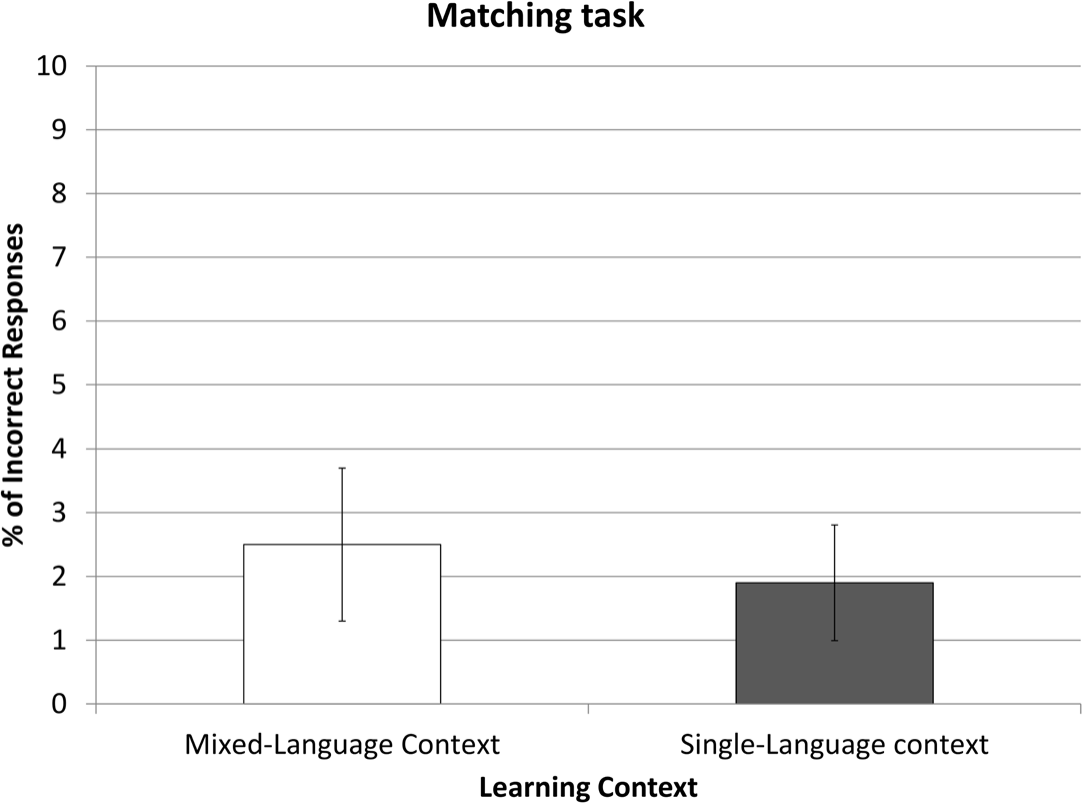
Error Rates in the Matching task in Experiment 1. Percentage of incorrectly associated learned objects to its real associate. Error bars represent confidence intervals of 95%.

In the last task participants were asked about the pseudo-objects they learnt and their conceptual association with familiar objects. Both groups were very accurate in identifying which real (*Familiar Related*) objects matched the learnt (*Unfamiliar Old*) items (an average of 1.76 errors over 40), whilst variance remained small (SD = 0.88). No significant differences in error rates were found between the SLC group and the MLC group (*t*(48) = .78, *p*>.44). Hence, the learning performance and the identification of meaning-related real-life objects were not hindered (or improved) by language context of the learning phase (see Fig 3).

### Interim summary

We tested the potential differences in learning, consolidation and integration of new information in adult balanced bilinguals that could be caused by language mixing during new information acquisition as compared to a monolingual learning context in which a single language is used. None of the measures obtained supported the idea that participants in a single-language learning context outperform those in a mixed-language context. No significant differences were observed in the measures associated to the learning phases or in the subsequently obtained direct and indirect indices of memory consolidation.

Given that the motivation of this study was to test a situation occurring in formal schooling, in Experiment 2 two groups of bilingual children attending a bilingual school were tested. Based on the findings from Experiment 1, and considering that language switching has been shown to occur spontaneously in very young children too (e.g., [26]) as well as recent evidence suggesting that the acquisition of a new skill is relatively similar for children acquiring it in a single-language and in multilingual learning contexts (e.g., [27]), we did not expect any specific advantage for the SLC as compared to the MLC group.

## Experiment 2: Children

### Methods

#### Ethics Statement

All the children enrolled in the experiment provided parental written informed consent. Prior to the experiment, parents received an informative letter and they signed the written informed consent form. They were appropriately informed regarding the basic procedure of the experiment, according to the ethical commitments established by the BCBL Scientific Committee and by the BCBL Ethics Committee that approved the experiment (Approval date: 19/03/2014; Approval reference: 19314J).

#### Participants

Fifty children (mean age 11.44, 31 females) who were attending a Basque-Spanish bilingual-immersion program on a 50–50% exposure basis since age 3 took part in the experiment. Children were randomly assigned to the SLC and MLC groups, and mimicking the procedure used with adult participants in Experiment 1, a series of control tasks was employed to validate the between-group matching. Pairwise comparisons showed that children who were randomly assigned to the SLC and MLC groups did not differ in age, gender, IQ, or the magnitude of the flanker and Simon effect. Also, their proficiency in Basque and Spanish evaluated on the basis of a 30-item multilingual picture naming test did not differ significantly (see [39]). In sum, the two groups did not differ in any of the dimensions tested (all *p*s.>.26, see Table 3.).

**Table 3:** Controlled variables in the children samples tested in Experiment 2.

	Age (years)	Gender (females)	IQ (correct responses)	Flanker Effect (ms)	Simon Effect (ms)	Spanish Vocabulary (out of 30)	Basque Vocabulary (out of 30)
MLC	11.32 (0.77)	56%	19.08 (2.74)	42 (70)	25 (58)	29.96 (0.2)	26.36 (1.91)
SLC	11.56 (1.05)	72%	20.12 (3.26)	35 (73)	51 (60)	29.92 (0.31)	26 (3.87)
p-value	0.31	0.24	0.26	0.68	0.21	0.57	0.62

Means are presented together with the standard deviations (in parentheses).

#### Materials and Procedure

Materials and procedures were identical to those used in Experiment 1.

## Results

### Learning (Test A and Test B)

In Test A, participants’ error rates did not differ between language contexts (*t*(48) = -.93, *p*>.36), showing similar learning curves across groups. In a similar vein, the two groups did not differ in the number of items seen during the learning phase (including the number of repeated items due to the repetition of a block after a given mistake) (*t*(48) = -.88, *p*>.38; see Table 4).

**Table 4:** Mean reaction times and error rates for all the experimental tasks used in Experiment 2.

		Learning—Test			Old-New judgment task										Matching
		Test A		Test B	Unfamiliar Old		Familiar New		Familiar Related		Unfamiliar New		False Memory Effect		
		Items seen	Error Rate	Error Rate	RTs	Error Rate	RTs	Error Rate	RTs	Error Rate	RTs	Error Rate	RTs	Error Rate	Error Rate
MLC	Mean	89.44	5.8	2.1	764	5.5	712	0.41	739	1	752	1.01	27	0.6	11.9
	SD	9.58	6.07	3.73	85	6.08	113	0.95	112	2.04	120	1.46	42	1.82	8.21
SLC	Mean	92	7.5	1.1	776	5.87	728	1.31	757	1.92	756	1.14	27	0.61	10.1
	SD	10.89	6.81	1.92	119	5.62	131	2.19	129	2.48	141	2.28	42	3.21	7.23

In Test B, no significant differences between the SLC and the MLC groups were found (*t*(48) = .24, *p*>.24).

#### Old-New judgment task

Following the same rationale as in Experiment 1, we first compared groups according to their responses to the *Unfamiliar Old* items (i.e., the items requiring an “Old” response). Response times did not differ between learning contexts (*t*(48) = -.42, *p*>.68), and in the same vein, the two groups did not differ in accuracy in the *Unfamiliar Old* condition (*t*(48) = -.23, *p*>.82).

**Fig 4:**
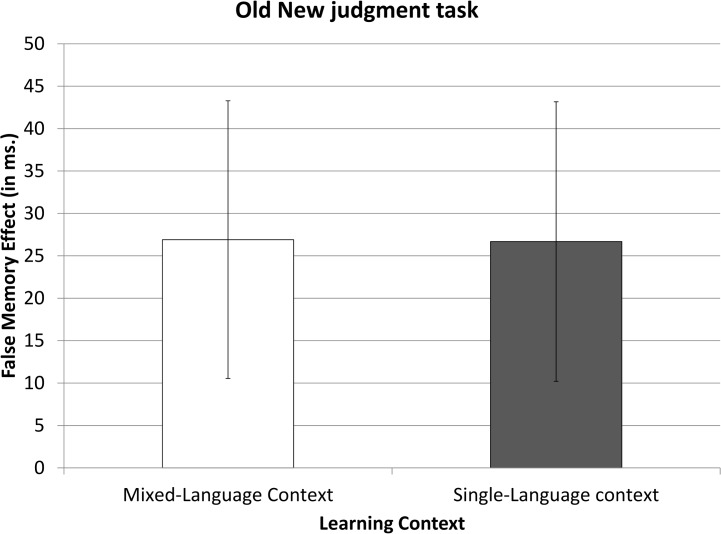
False memory effect in the Old-New judgment task in Experiment 2. This effect results from the subtraction of the reaction times to the Familiar New items from the Familiar Related items. Error bars represent confidence intervals of 95%.

Then, an ANOVA was conducted including all the conditions requiring a “New” response (i.e., *Unfamiliar New*, *Familiar New* and *Familiar Related*, in the same way as in Experiment 1, following a 3*2 (Condition*Learning Context) design (see Fig 4). The ANOVA on the RT data showed a main effect of Condition (*F*(2,96) = 13,50, *p*<.01), but just as in Experiment 1, the main effect of Learning Context was not significant (*F*(1,48) = .14, *p*>.71) and the interaction between the two factors was not significant (*F*(2,96) = .65, *p*>.53). The ANOVA on error data revealed that the main effect of Condition was not significant (*F*(2,98) = 1.52, *p*>.22). The main effect of Learning Context was not significant (*F*(1,48) = 2.90, *p*>.1), nor was the interaction between the two factors (*F*<1).

Next, we focused on the difference between responses in the *Familiar Related* and *Familiar New* conditions (i.e., the false memory effect). RTs were significantly shorter in the Familiar New than in the Familiar Related condition (720 ms vs. 748 ms, respectively; *t*(49) = 4.88, *p*<.01). Importantly, the magnitude of the false memory effect was highly similar in the SLC and MLC groups (an effect of 27 ms in both groups; *t*<1).

Finally, as in Experiment 1, mean RTs associated with the *Unfamiliar New* items and those associated with the *Familiar New* and *Familiar Related* items were compared, testing for a familiarity effect. Unfamiliar New items were responded to significantly more slowly than Familiar New items (754 ms and 720 ms, respectively; *t*(49) = 4.22 *p*<.01), but not significantly so from Familiar Related items (748 ms; *t*<1).

#### Matching task

**Fig 5:**
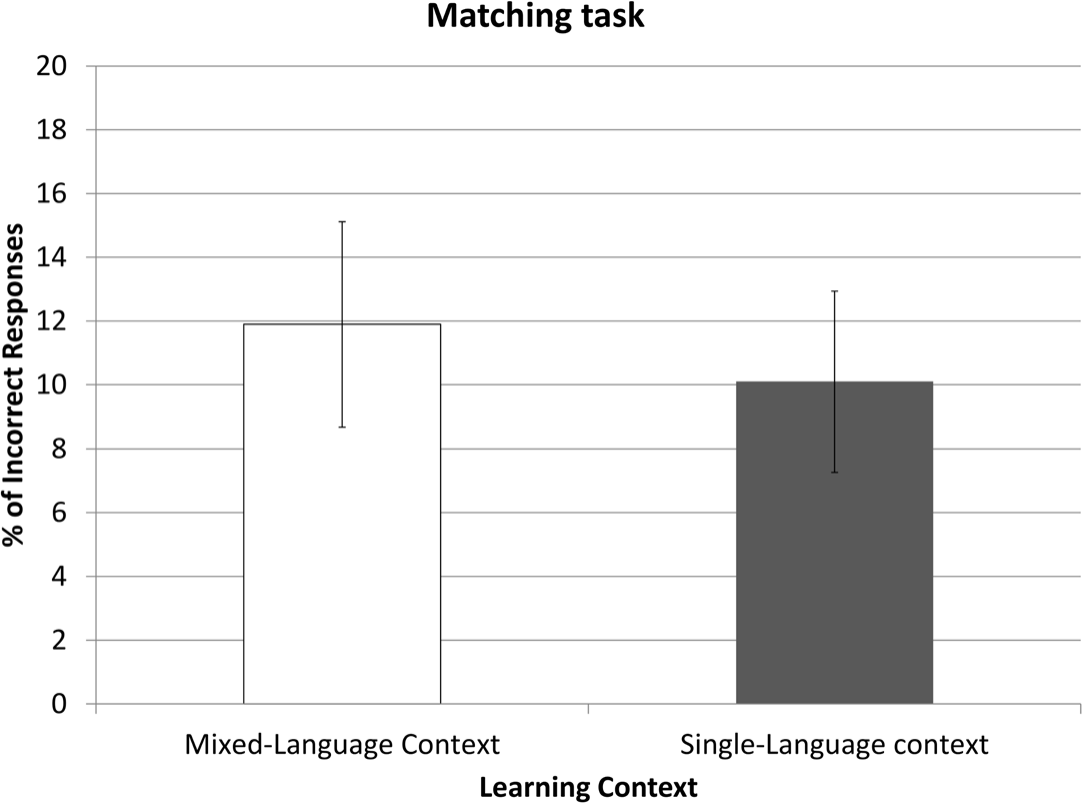
Error Rates in the Matching task in Experiment 2. Percentage of incorrectly associated learned objects to its real associate. Error bars represent confidence intervals of 95%.

Both SLC and MLC groups showed markedly high accuracy rates (see Fig 5), with a mean error rate of 11% (SD = 7.71) (an average of 35.6/40 hits). There was no significant difference in accuracy between Learning Contexts (*t*(48) = .82, *p*>.42).

### Interim summary

Results from Experiment 2 fully replicated those obtained in Experiment 1. There were no significant differences between the single-language and the mixed-language learning context in the learning trends or in direct and indirect measurements of learning and consolidation. These results show that bilingual children acquire concepts equally efficiently irrespective of the language context (separate or mixed) used for tuition. Thus, these results provide evidence against the one subject-one language rule commonly applied in bilingual educational contexts.

## General Discussion

The aim of this study was to test whether mixing languages during the process of learning new concepts hinders concept acquisition and the consolidation in semantic memory of the learnt concepts. Bilingualism has long been considered a delaying factor in child development, and its possible detrimental impact in different contexts such as schooling and parenting has been feared. Thanks in part to scientific evidence; such misconceptions have been gradually changed. In contrast to earlier studies showing significant differences in vocabulary size, word production and comprehension between bilinguals and monolinguals [54–55], recent studies have suggested that these differences are not reliable and that they do not speak for a ‘bilingual disadvantage’ [56–57]. Hence, bilingual children appear to reach developmental and linguistic milestones in their two languages at a similar pace and their developmental trajectory does not dramatically differ from that of monolingual children. Unfortunately, the belief that mixing languages in the process of learning may be detrimental for the learning process is an ulcer that still needs to be extirpated on the basis of solid scientific data.

Here we simulated essential stages of the process involved in learning new concepts based on the presentation of novel objects paired with definitions while manipulating the number of languages used during the learning phase (mixed-language or single-language learning contexts). In Experiment 1, adult balanced bilinguals were tested in a series of experimental paradigms aimed at exploring 1) differences in the learning phase depending on the number of languages used during the process, and 2) differences in the consolidation and integration of the learnt concepts in semantic memory as a function of the number of languages used during learning. Importantly, none of the indices obtained favored the single-language over the mixed-language context. Hence, we found negligible differences between the two contexts during concept acquisition, showing that language mixing does not represent any additional difficulty for concept learning in balanced bilingual adults. More importantly, indirect measures of learning and integration in semantic memory, as measured by the false memory effects evoked by the familiar objects that were semantically related to the objects learnt also showed parallel (and successful) integration in the two learning contexts.

Finally, a direct measure of learning and integration based on an explicit association between the learnt novel objects and existing known objects overlapping in their features and use with the former also showed no differences between groups. Thus, results from Experiment 1 suggest that a multilingual learning context in which two languages are mixed during instruction does not have a negative impact on the learning process itself, nor does it hinder the connection of learnt concepts with pre-existing semantic representations. Together, these data provide evidence against assumptions in support of the one subject–one language rule in formal schooling.

In Experiment 2, we directly explored whether the same conclusions would stand in the case of bilingual children attending a bilingual school where two languages are used on a daily basis (but only one language is used for instruction in a given subject). Overall, results from the children samples tested in Experiment 2 closely replicated the outcomes of Experiment 1 (adults). There were no significant differences in the learning trends as a function of the number of languages used during concept acquisition, and both groups of children (SLC and MLC) performed equally well in the experimental paradigms designed to explore the extent to which the learnt concepts had been integrated in semantic memory. Taken together, results from Experiment 2 demonstrate that the simultaneous use of two languages rather than one during concept learning does not increase learning difficulty in balanced bilingual children attending bilingual schools.

The absence of differences in the acquisition of new concepts by language-mixing and single-language methodologies could be effectively interpreted as a consequence of automatic and effortless mental translation processes. The transition from one language to another takes place within a few tens of milliseconds in the case of balanced bilinguals, and there is now tangible evidence that the two languages of a bilingual individual are active even if only one of them is required for the task at stake [32–38]. According to this view, one could tentatively suggest that learners in both mixed-language and single-language contexts activate the lexical representations from their two languages in parallel, irrespective of the number of languages involved in the learning process, thus leading to highly similar effects in the two learning contexts (given the fact that access to semantic representations is equally effective in the two languages). In the same vein, models of bilingual lexico-semantic organization such as the Revised Hierarchical Model (RHM; see [44, 45, 58]) suggest that at sufficiently high levels of proficiency, bilingual individuals access language-independent semantic representations efficiently regardless of input language.

To date, the very few studies systematically exploring the impact of monolingual vs. bilingual education have mainly focused on the differences between bilingual schooling programs (i.e., bilingual education) and fully monolingual schooling programs (see [59]). Different meta-analyses have shown that bilingual education is consistently superior to fully monolingual approaches for second language learning (e.g., [60]). However, these studies exclusively focused on the benefits associated to bilingual schooling programs in which the two languages are not intermixed within a single-subject context (i.e., programs following the one subject-one language rule), and little was known about the differential impact of using one vs. two languages simultaneously for tuition in bilingual schools. The current study is, to the best of our knowledge, the first to investigate this issue, and results support the view that young and older balanced bilinguals learn in a similar manner when immersed in single-language and mixed-language learning contexts.

These results invalidate the premises of the one subject–one language rule and indicate that comparable concept acquisition and integration can be achieved by balanced bilingual learners irrespective of the number of languages used during tuition. It is worth noting that a mixed-language learning context is more akin than a single-language learning context to the linguistic reality of multilingual societies where more than one language are used on a regular basis (i.e., spontaneous language switching). Besides, the simultaneous use of two languages during learning increases the likelihood of balanced exposure, promoting parallel development of linguistic abilities. (Note that this may indeed represent a benefit for children who are still developing their linguistic skills).

The current study only focused on balanced bilingual adults and children. Future research will determine whether the same results can be obtained with samples of non-balanced bilinguals who are dominant in one of their languages, given that the cognitive effort associated with mental translation is different in balanced and imbalanced bilinguals (e.g., [40,61]). We acknowledge that future studies should also test whether parallel results could be obtained with different language combinations. Basque and Spanish are markedly different at the lexical and syntactic level, but they share phonology and orthography to a large extent (i.e., they both are alphabetic languages with similar grapheme-to-phoneme mappings). Hence, it would be interesting to explore the consequences of language mixing during learning in language combinations with different degrees of linguistic distance. Furthermore, and in contrast to naturalistic bilingual code-switching where words of the two languages could be blended together during natural speech in a single sentence, for the sake of simplicity we constrained our materials so that language mixing occurred at the whole-sentence level. Further research in needed in order to elucidate whether similar results could be obtained using different forms of code-switching (e.g., within-sentence). Finally, it is worth highlighting that the type of information underpinning learning in the current experiment was (a) simple (so that it would be compatible with learning by school children), and (b) exclusively based on semantic extension (i.e., based on the functional connection of novel objects with previously known ones). Follow up studies are required to examine whether the acquisition of expert knowledge and/or concepts disconnected from pre-established representations is also impervious–or perhaps significantly improved–by language mixing.

In sum, this study shows that there is currently no evidence supporting educational practices that exclude mixing languages during the learning of new concepts. These results establish that the final stage of semantic integration (which we consider to be the goal of explicit concept learning) is similarly achieved irrespective of the number languages used to present or define the new concepts. In other words, and at least in a context where there is good and equal mastery of two languages, language mixing is not detrimental to the learning process and does not incur quantifiable learning or integration costs as compared to single-language context learning. In the absence of any negative effect of language mixing, of a delay or poorer learning performance, only the positive outcomes of language mixing can be considered. Firstly, parallel exposure to and use of two languages represent new opportunities for the development of linguistic competence. Secondly, the simultaneous use of two languages makes the learning experience more ecological with respect to the way in which bilingual societies naturally function.

## Supporting Information

S1 Appendix Definitions in Spanish (red) and in Basque (green) used in the learning phase for the SLC and MLC groups. The items are numbered in the same order as in S2 Appendix.
(PDF)

S2 Appendix Pictures used in Experiments 1 and 2. The items are numbered in the same order as in S1 Appendix.
(PDF)
