# Supplementary material for: Mixing Languages during Learning? Testing the One Subject—One Language Rule
Source: PLoS One. 2015 Jun 24;10(6):e0130069. doi: 10.1371/journal.pone.0130069 (PMC4479465; doi:10.1371/journal.pone.0130069)
Supplement: S1 Appendix — The items are numbered in the same order as in S2 Appendix. (PDF) [file pone.0130069.s001.pdf]

S1 Appendix: Definitions in Spanish (red) and in Basque (green) used in the learning phase for the SLC and MLC groups. The items are numbered is the same order as in S2 Appendix.

Item	Definition 1 for SLC	Definition 2 for SLC
1	Siempre apunta al norte.	Los exploradores lo usan para no perderse.
2	Puede escribir de distintos colores.	Tiene tinta dentro.
3	Se usa en el tendedero para sujetar la ropa.	Puede ser de madera o plástico.
4	Corta el pelo de la piel.	Se usa después de ponerse espuma.
5	Sujeta los pantalones a la cintura.	Se puede apretar o aflojar.
6	Lo usan los médicos para vacunar.	Se usa para sacar sangre.
7	Está en los zapatos.	Sujeta el calzado al pie con un nudo.
8	Se usa para lavarse los dientes tres veces al día.	Se le pone pasta.
9	Refleja lo que tiene delante.	Lo usamos para mirarnos en el baño.
10	Es una joya que se pone en el dedo.	Lo llevan los casados.
11	Agranda las cosas al mirar por su cristal.	Lo usan los detectives.
12	Se golpea con una raqueta.	Bota contra el suelo.
13	Se guarda en el bolsillo.	Abre las cerraduras de las puertas.
14	Se lleva de excursión.	El agua de dentro la mantiene fresca.
15	Se lleva en la cartera y se usa para pagar.	Saca dinero de los cajeros.
16	Se apunta a la televisión para cambiar de canal.	Sube y baja el volumen.
17	Las chicas se lo ponen en las orejas.	Se colocan en un agujero.
18	Dispara balas.	Los policías tienen.
19	Funciona con pilas.	Hace luz al encenderla en la oscuridad.
20	Se mete dinero dentro para ahorrar.	Para sacar el dinero hay que romperlo.
21	Está muy afilado.	Se usa para comer carne.
22	Se usa con hilo para coser.	Se empuja con un dedal.
23	Se usa en el parchís.	Rueda por encima de la mesa y te da un número.
24	Se sopla con la boca y hace ruido.	Lo usan los árbitros.
25	Está en la bragueta.	Se sube para cerrarlo y se baja para abrirlo.
26	Se rellena de vino con una botella.	Se usa para beber.
27	Se usa para llamar a amigos que estén lejos.	Tiene teclas que se pulsan.
28	Se enciende con un mechero.	Se rellena de tabaco y se fuma.
29	Se mueve con la mano para manejar el ordenador.	Está cerca del teclado.
30	Saca el líquido de las naranjas.	Se usa en el desayuno para hacer zumos.
31	Los bebés se lo ponen en la boca.	Se chupa y ayuda a dejar de llorar.
32	Se mete en la mochila para llevarlo al colegio.	Sirve para guardar lápices.
33	Se usa con pintura.	Los artistas lo usan para hacer cuadros.
34	Nos permite medir cosas en la escuela.	Sirve para hacer líneas rectas.
35	Se pone en las tartas de cumpleaños.	Tiene una llama que se apaga soplando.
36	Se pone en la muñeca.	Tiene números que te dan la hora.
37	Se echa al buzón con un sello.	Puedes poner una carta dentro.
38	Se ponen frente a los ojos para corregir la vista.	Se sujetan en la nariz.
39	Se gasta al escribir en la pizarra.	Lo que escribe se limpia con un borrador.
40	Sirve para golpear clavos y clavarlos.	Lo usan los carpinteros.

Item	Definition 1 for MLC	Definition 2 for MLC
1	Beti iparraldea erakusten du.	Los exploradores lo usan para no perderse.
2	Kolore desberdinetan idatzi dezake.	Tiene tinta dentro.
3	Se usa en el tendedero para sujetar la ropa.	Egurrezkoa edo plastikozkoa izan daiteke.
4	Azaleko ileak mozteko erabiltzen da.	Se usa después de ponerse espuma.
5	Galtzak gerrira eusten ditu.	Se puede apretar o aflojar.
6	Lo usan los médicos para vacunar.	Odola ateratzeko erabiltzen da.
7	Oinetakoetan dago.	Sujeta el calzado al pie con un nudo.
8	Egunean hirutan hortzak garbitzeko erabiltzen da.	Se le pone pasta.
9	Aurrean daukana isladatzen du.	Lo usamos para mirarnos en el baño.
10	Es una joya que se pone en el dedo.	Ezkonduek daramate.
11	Bere kristaletik begiratzean gauzak haunditzen ditu.	Lo usan los detectives.
12	Erraketarekin ematen zaio.	Bota contra el suelo.
13	Poltsikoan gordetzen da.	Abre las cerraduras de las puertas.
14	Txangoetara eramaten da.	El agua de dentro la mantiene fresca.
15	Se lleva en la cartera y se usa para pagar.	Kutxazainetatik dirua ateratzeko balio du.
16	Telebistako kateak aldatu ditzake.	Sube y baja el volumen.
17	Neskek belarrietan erabili ohi dute.	Se colocan en un agujero.
18	Dispara balas.	Poliziek erabiltzen dute.
19	Funciona con pilas.	Iluntasunean piztean argitu egiten du.
20	Se mete dinero dentro para ahorrar.	Dirua ateratzeko puskatu egin behar da.
21	Está muy afilado.	Haragia jateko erabiltzen da.
22	Se usa con hilo para coser.	Titare batekin bultzatzen zaio.
23	Partxisean erabiltzen da.	Rueda por encima de la mesa y te da un número.
24	Ahoarekin putz egitean soinua egiten du.	Lo usan los árbitros.
25	Está en la bragueta.	Isteko igo eta irekitzeko jaitsi egiten da.
26	Se rellena de vino con una botella.	Edateko erabiltzen da.
27	Urruti dauden lagunei deitzeko erabiltzen da.	Tiene teclas que se pulsan.
28	Se enciende con un mechero.	Tabakoz bete eta erre egiten da.
29	Eskuarekin mugitzen da ordenagailua erabiltzeko.	Está cerca del teclado.
30	Saca el líquido de las naranjas.	Gosarietan zukuak egiteko erabiltzen da.
31	Ume txikiek ahoan sartzen dute.	Se chupa y ayuda a dejar de llorar.
32	Se mete en la mochila para llevarlo al colegio.	Arkatzak gordetzeko erabiltzen da.
33	Se usa con pintura.	Artistek margolanak egiteko erabiltzen dute.
34	Nos permite medir cosas en la escuela.	Marra zuzenak egiteko balio du.
35	Urtebetetze tartatan jartzen da.	Tiene una llama que se apaga soplando.
36	Eskumuturrean eramaten da.	Tiene números que te dan la hora.
37	Se echa al buzón con un sello.	Gutun bat sartu dezakezu barruan.
38	Se ponen frente a los ojos para corregir la vista.	Sudurrean heltzen dira.
39	Arbelan idazterakoan agortu egiten da.	Lo que escribe se limpia con un borrador.
40	Sirve para golpear clavos y clavarlos.	Arotzek erabiltzen dute.
