# Supplementary material for: Mixing Languages during Learning? Testing the One Subject—One Language Rule
Source: PLoS One. 2015 Jun 24;10(6):e0130069. doi: 10.1371/journal.pone.0130069 (PMC4479465; doi:10.1371/journal.pone.0130069)
Supplement: S2 Appendix — The items are numbered in the same order as in S1 Appendix. (PDF) [file pone.0130069.s002.pdf]

S2 Appendix: Pictures used in Experiments 1 and 2. The items are numbered in the same order as in S1 Appendix.

Item	Unfamiliar Old	Familiar Related	Unfamiliar New	Familiar New
1	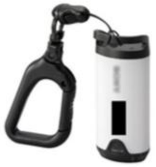	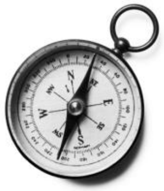	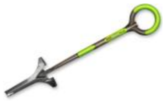	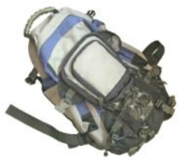
2	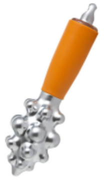	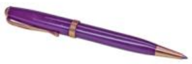	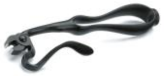	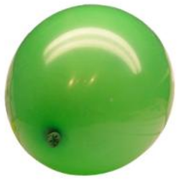

3	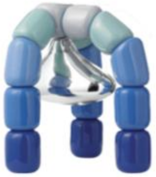	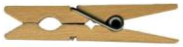	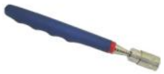	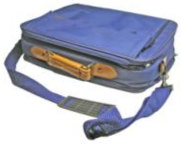
4	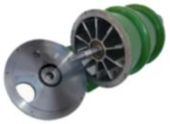	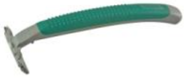	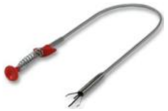	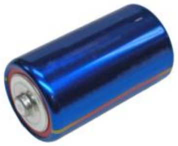
5	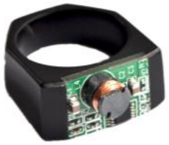	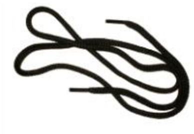	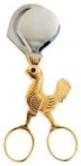	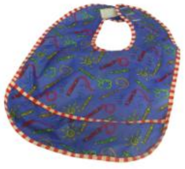

6	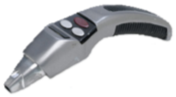	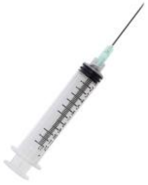	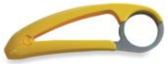	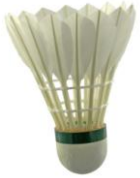
7	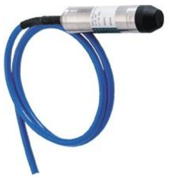	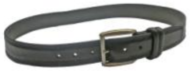	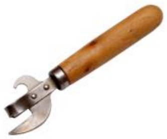	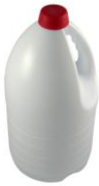
8	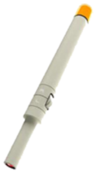	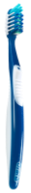	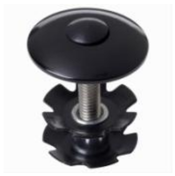	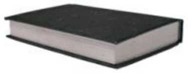

9	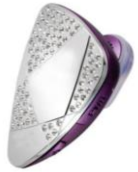	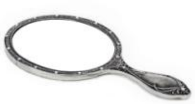	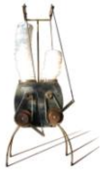	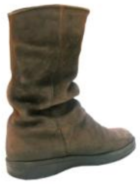
10	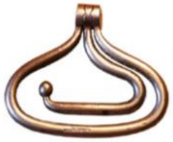	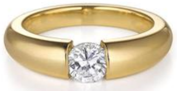	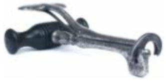	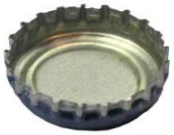
11	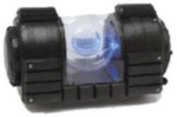	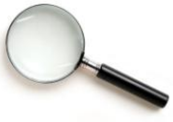	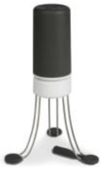	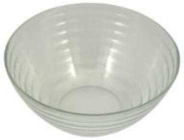

12	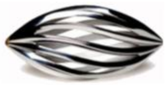	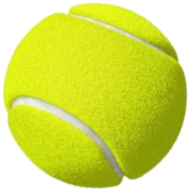	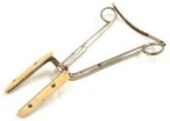	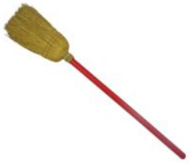
13	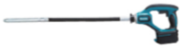	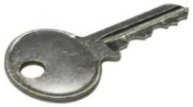	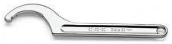	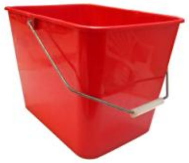
14	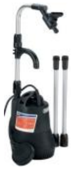	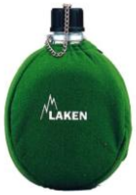	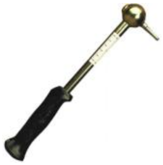	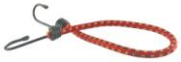

15	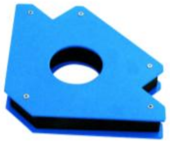	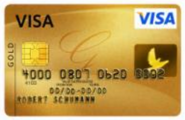	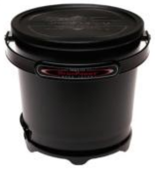	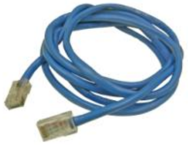
16	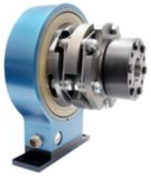	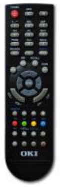	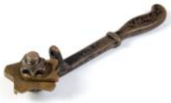	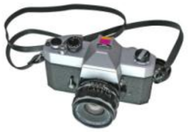
17	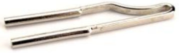	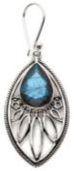	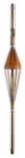	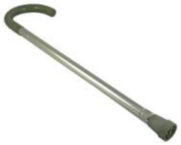

18	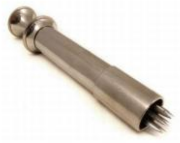	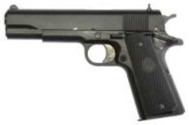	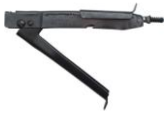	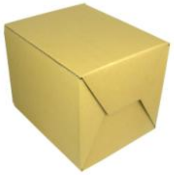
19	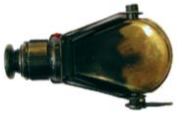	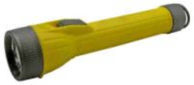	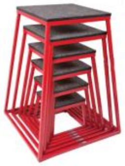	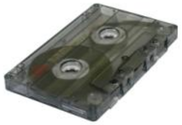
20	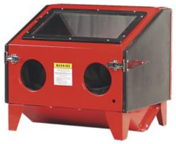	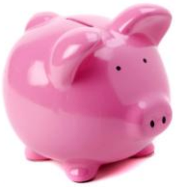	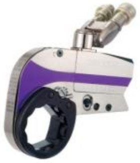	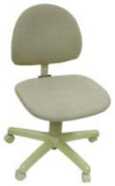

21	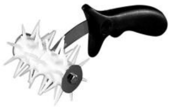	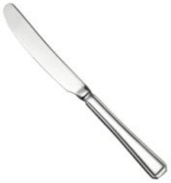	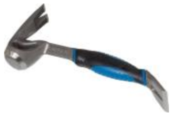	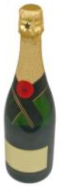
22	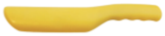	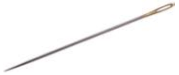	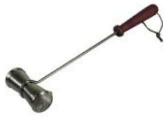	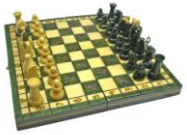
23	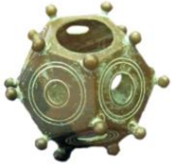	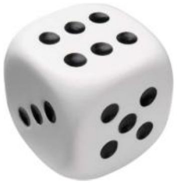	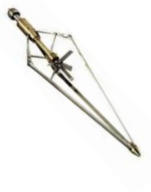	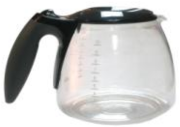

24	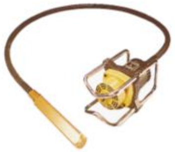	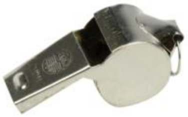	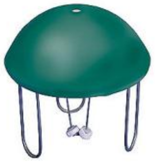	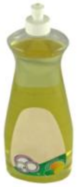
25	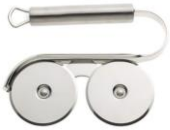	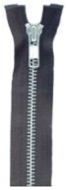	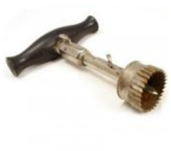	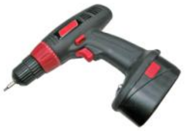
26	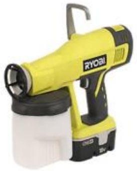	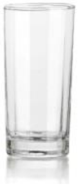	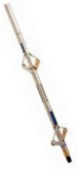	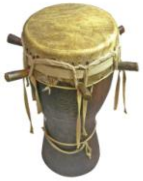

27	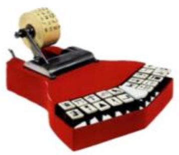	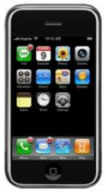	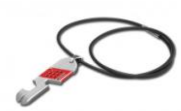	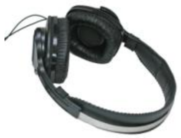
28	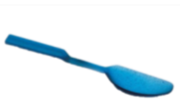	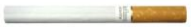	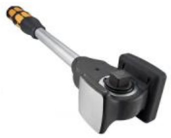	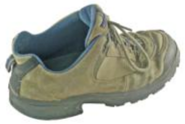
29	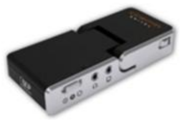	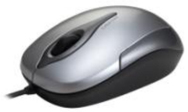	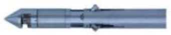	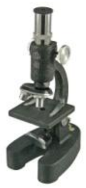

30	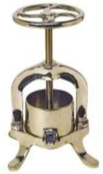	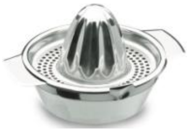	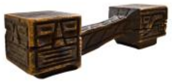	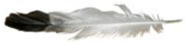
31	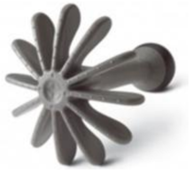	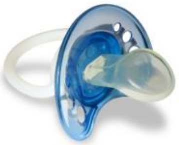	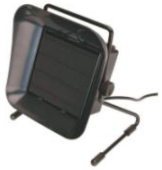	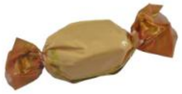
32	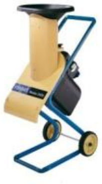	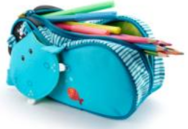	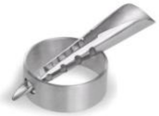	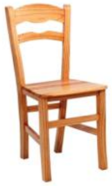

33	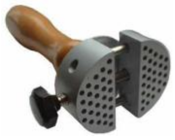	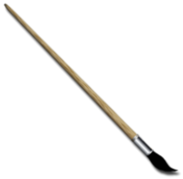	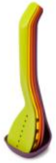	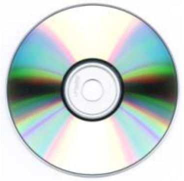
34	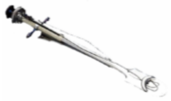	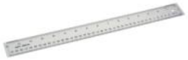	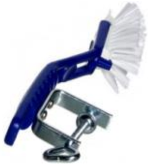	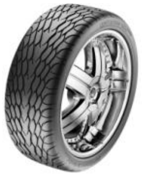
35	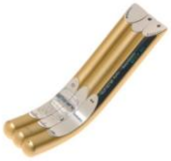	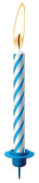	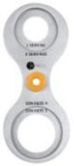	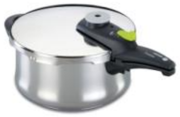

36	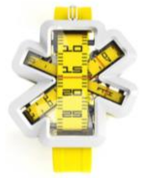	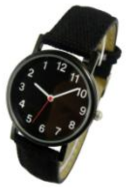	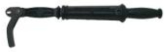	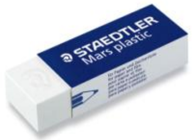
37	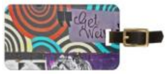	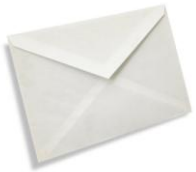	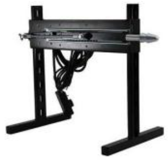	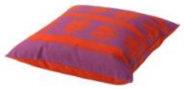
38	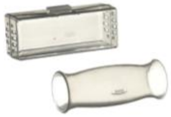	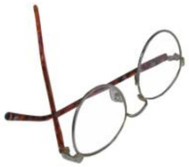	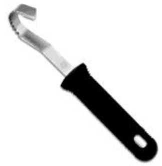	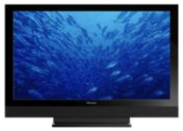

39	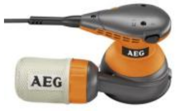	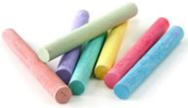	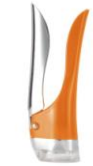	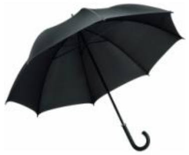
40	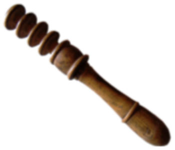	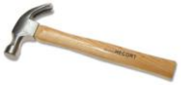	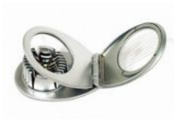	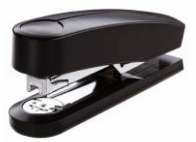
